# Late diagnosis of systemic lupus erythematosus and antiphospholipid syndrome in an older woman with psychosis: a case report and review of the literature

**DOI:** 10.1002/ccr3.1185

**Published:** 2017-09-26

**Authors:** Pablo Rodrigo Montero‐Olvera, Roberto Berebichez‐Fridman, Lorena Velázquez‐Álvarez, Juan Rogelio Ríos‐Morales, Manuel Alejandro Rodríguez‐Guiza

**Affiliations:** ^1^ School of Medicine Faculty of Health Sciences Anahuac University Mexico North Campus Huixquilucan State of Mexico Mexico; ^2^ Neurology Service Hospital General de México “Dr. Eduardo Liceaga” Mexico City Mexico; ^3^ Psychiatry Section Hospital Central Militar Mexico City Mexico; ^4^ Military School for Sanitation Graduates Military Center of Health Sciences Mexico City Mexico

**Keywords:** Antiphospholipid syndrome, dementia, psychogeriatrics, psychosis, systemic lupus erythematosus

## Abstract

In older adults with first‐time psychiatric manifestations, physical disorders such as systemic lupus erythematosus (SLE) and antiphospholipid syndrome (APS) must be considered, even in patients with cognitive dysfunction and/or dementia. Here, we describe the case of a 77‐year‐old woman with psychosis who was later diagnosed with SLE and APS.

## Introduction

Systemic lupus erythematosus (SLE) is a potentially fatal autoimmune disease that remains frequently underdiagnosed due to the heterogeneity of its symptoms. SLE is characterized by the production of autoantibodies that cause pathogenic manifestations such as fever, fatigue, alopecia, sun sensitivity, Raynaud's phenomenon, butterfly rash, livedo reticularis, oral ulcers, arthritis, and serositis in affected patients. These autoantibodies may be present up to a decade prior to diagnosis and the development of overt physical manifestations 1. The prevalence of SLE varies in accordance with sex and race, being especially prevalent in women between 15 and 50 years old (163/100,000). Prevalence ranges from 40 cases per 100,000 in Caucasians to 200/100,000 in African Americans, while prevalence in Hispanics has been reported as 126/100,000 2, 3.

Antiphospholipid syndrome (APS) is accompanied by the presence of laboratory biomarkers and clinical manifestations such as thrombosis, thrombocytopenia, and recurrent fetal loss. Criteria for APS diagnosis do not include cerebral ischemia, dementia, or psychosis 4; however, evidence suggests that both SLE and APS are associated with significant morbidity owing to neurological disturbances caused by vascular damage to the central nervous system (CNS), which can be the presenting feature of the disease 5, 6.

Symptoms of psychosis such as hallucinations, delusions, and disorganized behavior are included within the diagnostic criteria of SLE 10. Approximately 2–11% of patients with SLE and APS present with such symptoms, although diagnosis remains challenging when other clinical features are mild 10. The American College of Rheumatology (ACR) identifies 19 “case definitions” of neuropsychiatric conditions, among which psychosis and cerebrovascular disease are present 10.

Secondary antiphospholipid syndrome (SAPS) occurs when both SLE and APS overlap, complicating the identification of the true underlying mechanisms of the disease. SAPS has been identified more frequently in older adults, in whom cognitive symptoms are more evident. In fact, SAPS is so common that some clinicians prefer the term “SLE‐associated APS”: APS is present in 30–40% of patients with SLE, 30–70% of whom go on to develop SAPS 7, 8. Previous reports have indicated that neurocognitive dysfunction associated with SLE and APS occurs primarily in patients with elevated immunoglobulin G (IgG) and IgA anticardiolipin levels 9.

## Case Report

The patient of the present case was a 77‐year‐old woman who was first hospitalized at the age of 65 years owing to mystical religious delusions, psychomotor agitation, and physical/verbal aggression. She had escaped her home, returning 8 days later wearing different clothes and exhibiting persistent symptoms of psychosis. Dermabrasions were also observed on her arms. Noncontrast computed tomography (CT) and electroencephalography (EEG) findings were normal. Throughout her hospitalization, she experienced visual and auditory hallucinations, as well as delusional and persecutory delusions, which she described as the main reasons for leaving her home. She was diagnosed with acute psychotic disorder and discharged on risperidone after 10 of hospitalization. Response to treatment was irregular and incomplete.

Upon discharge, family members reported the presence of psychotic symptoms and disorganized conduct: The patient had blocked the windows to her room and had begun waking up early to avoid being seen eating. Family members also began to notice deformities on her hands, but did not mention this at the time. Thus, no other diagnosis could be established.

**Figure 1:**
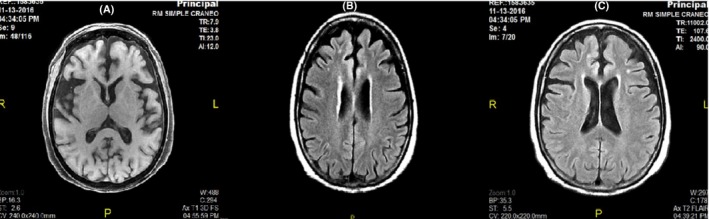
Axial‐brain MRI T1 FLAIR sequence that shows cortico‐subcortical atrophy of frontotemporal predominance and compensatory ventriculomegaly (A). T2 FLAIR sequence shows focal hyperintense subcortical lesions in frontal lobe and periventricular area, <1 cm in diameter, consistent with small‐vessel disease (B, C).

Six months after her initial hospitalization, she was re‐admitted due to soliloquy, isolation, abnormal mannerisms, visual/auditory hallucinations, and physical and verbal aggressiveness and compulsions related to cleanliness. These compulsions included washing her face with boiling water, causing second‐degree burns to her forehead. She developed mystic, religious, and persecutory delusions; sleep cycle alterations; and hyporexia. Brain magnetic resonance imaging (MRI) revealed the presence of small‐vessel disease with frontotemporal predominant cortico‐subcortical atrophy (Fig. 1). Doppler ultrasonography of the carotid arteries revealed only mild atherosclerosis, while EEG revealed no abnormal findings.

She was diagnosed with vascular dementia and started on memantine, acetylsalicylic acid, haloperidol, quetiapine, and trihexyphenidyl. She was discharged from the hospital on the 4th day.

Ten months following her second hospitalization, she was once again hospitalized due to persistent psychotic symptoms. Family members attributed her behavior to a previous medication switch from quetiapine to olanzapine based on inadequate response to the former. She was discharged on clonazepam, quetiapine, memantine, and donepezil.

During one of her scheduled appointments, routine laboratory values were obtained. Venereal disease research laboratory (VDRL) test results were positive, following which a more extensive workup was requested.

The complete laboratory test results are shown in Table 1.

**Table 1:** Laboratory Results

Parameter	Result	Reference Values	Units
Leukocytes	3.39	3.56–10.30	10^3^/*μ*L
Erythrocytes	4.06	3.87–5.44	10^6^/*μ*L
Hemoglobin	13.1	11.70–16.30	g/dL
Hematocrit	40.8	35.40–49.40	%
Mean corpuscular volume (MCV)	100.6	83.30–100.00	fL
GSR (Globular sedimentation rate)	16	0–30	mm/h
Mean cell hemoglobin content (MCHb)	32.2	26.8–33.2	pg
Platelets	217	157–431	10^3^/*μ*L
Lymphocytes %	18.2	15.5–48.5	%
Neutrophils %	60.2	39.6–76.1	%
Monocytes %	14.7	3.4–10.1	%
Eosinophils %	5.1	0.3–5.5	%
Basophils %	0.7	0.0–1.4	%
LUC%	1.000	0.0–1.4	%
Lymphocytes #	0.62	0.99–3.24	10^3^/*μ*L
Neutrophils #	2.04	1.71–6.48	10^3^/*μ*L
Monocytes #	0.5	0.19–0.71	10^3^/*μ*L
Eosinophils #	0.17	0.02–0.32	10^3^/*μ*L
Basophils #	0.02	0.00–0.09	10^3^/*μ*L
Large unstained cells number (LUC)	0.03	0.000–0.400	10^3^/*μ*L
Red cell distribution width (RDW)	13.7	12–17.7	%
Mean platelet volume	9	8–12	fL
C3	48	88–165	mg/dL
C4	8.5	14–44	mg/dL
Rheumatoid Factor	<9	0–12	UI/mL
Antinuclear antibodies			
Result	1:40 dilution	N/A	Negative
Positivity	(++++)	N/A	
Pattern	homogeneous	N/A	
Anti‐dsDNA	Negative 1:10	<20 Negative 20–30 Weak Positive 40–80 Mild Positive >80 Strong Positive	U
Ab. IgG ANTI RNP	2.46	<20 Negative 20–30 Weak Positive 40–80 Mild Positive >80 Strong Positive	U
Ab. IgG ANTI SS‐ (Ro)	61.27	<20 Negative 20–30 Weak Positive 40–80 Mild Positive >80 Strong Positive	U
Ab. IgG ANTI SS–B(La)	9.12	<20 Negative 20–30 Weak Positive 40–80 Mild Positive >80 Strong Positive	U
Ab. IgG ANTI CCP 3rd generation	11.11	<20 Negative 20–30 Weak Positive 40–80 Mild Positive >80 Strong Positive	U
Antiphospholipid antibodies			
Anticardiolipin antibodies IgG IgM	112 116	<20 Negative 20–30 Weak Positive 40–80 Mild Positive >80 Strong Positive	U
Lupus anticoagulant	Negative	Negative	NA
Clotting tests			
PT (Prothrombin time)	15.6	Sec	11–13 sec
PTT (Partial thromboplastin time)	29.45		25–35 sec
INR	1.18		0.8–1.1 sec

Diagnoses of SLE and APS were made based on laboratory and clinical findings. The diagnosis of SLE was based on the presence of leukopenia, arthritis, neurological signs, antiphospholipid antibodies, and hypocomplementemia. The patient was started on azathioprine, rivaroxaban, pulses of methylprednisolone, and two doses of rituximab, with adequate and complete response. No psychotic symptoms were observed or reported by the patient's family at her latest follow‐up.

## Discussion

According to the ACR, neuropsychiatric SLE (NPSLE) is defined as “neurologic symptoms of central, peripheral, and autonomic nervous systems, and psychiatric syndromes observed in patients with SLE, in which other causes have been excluded” 11.

NPSLE is rare in older adults, although the number of documented cases continues to increase (Table 2). Dennis et al. reported a series of five patients between 75 and 93 years of age diagnosed with NPSLE. Each patient had initially presented with confusion, dementia, and depression that preceded the diagnosis of NPSLE by several years. Psychosis was a common feature among all five patients 12.

**Table 2:** Documented cases

Patient	Presentation	Laboratories	Imaging	Initial Treatment	References
Female, 77 years ‐old (Present Case)	Mystical religious delusions, psychomotor agitation, verbal and physical aggression	Anticardiolipin IgG 112, IgM 116 ANA 1/160 Rheumatoid factor <12 C‐reactive protein: 3.48 C3: 0.57 C4: 0.1	Brain MRI showing cortico‐subcortical atrophy of frontotemporal predominance, compensatory ventriculomegaly, focal hyperintense subcortical lesions in frontal lobe and periventricular area, <1 cm in diameter, consistent with small‐vessel disease	Clonazepam, quetiapine, memantine, and donepezil	NA
Female, 49 years old	Persecutory delusions, reactive anxiety, and low mood. No signs of autoimmune disease	Lymphopenia, ANA 1/1280, dsDNA antibodies 1/60. RF, and ANTI‐ENA negative. CRP, ESR, and complement were normal	Brain MRI showing multiple hyperintensities in the subcortical white matter of the frontal and parietal lobes	Atypical neuroleptics	20
Female, 15 years old	Fever, dizziness, trouble speaking, blurred vision. Followed by dysphoria, delusions, and edema	Thrombocytopenia 40 x 10^9^/L, hypocomplementemia, positive ANA of 1:100, positive anti‐Rib‐p, anti‐SSA, and positive Coomb's test. APTT 89.7s. Positive anticardiolipin IgG, anti‐b2 IgG, and IgM	Brain MRI showing hemorrhage of the left frontotemporal lobe, right parietal lobe, right temporal lobe, and right cerebellar hemisphere; and thrombosis of the sagittal sinus and right transverse sinus	Methylprednisolone, quetiapine, and mannitol	21
Female, 47 years old	Persecutory delusions, delusions of reference, unveiling delusions, delusions of stealing ideas, and delusions of complex and bizarre substance accompanied by illusory and hallucinatory sensations	ANA 1:320, hypocomplementemia, thrombocytopenia	Brain MRI showing bilateral hyperintensities on T1‐weighted images and hypointensities on T2‐weighted images in the basal ganglia. T2 and FLAIR images showing hyperintensities in the subcortical white matter	Haloperidol for 4 years, followed by aripiprazole and flupentixol	22
Female, 50 years old	Schizophrenia‐like syndrome	Positive serological test for syphilis and positive Wassermann reaction, ANA 1:10.24, Rho‐positive antibodies present, anticardiolipin antibodies increased 33 U/mL (normal <10) for IgG, 17 U/mL (normal <6) for IgM	Brain CT showing infarction and foci of calcification	Corticoids, azathioprine, argininosuccinate synthetase, and haloperidol	23

The classification of NPSLE has undergone several revisions, beginning in 1999 with the ACR standardization of case definitions and nomenclature 13. The Systemic Lupus International Collaborating Clinics (SLICC) proposed that revised criteria include psychosis and cerebrovascular disease (among others), based on the frequency with which these symptoms are encountered 14.

NPSLE is estimated to occur in 20–75% of adult patients with SLE 15, 16. Currently, there is no single clinical, laboratory, or imaging test that can be used to differentiate patients with NPSLE from those with non‐NPSLE 17. However, previous studies have indicated that patients who present with neuropsychiatric manifestations associated with antiphospholipid antibodies (aPLs) may exhibit nonspecific multifocal white matter lesions or multiple sclerotic lesions localized within subcortical, periventricular areas, and in the corpus callosum on brain MRIs 18.

The antinuclear antibodies (ANA) test is positive in 95% of patients with SLE, although its false‐positive rate is 30%. Thus, its low specificity and positive‐predictive values render the ANA test insufficient for establishing a diagnosis of SLE. Additional laboratory and clinical characteristics such as anemia, thrombocytopenia, leukopenia, proteinuria or hematuria, and hypocomplementemia (C3 and C4) are therefore required for a definitive diagnosis 19.

Because aPLs (i.e., anticardiolipin antibodies) are commonly found in patients with SLE, false‐positive VDRL results are also common. APS occurs when both hypercoagulability and aPLs are present 19. At least one of the following aPLs must be present for a diagnosis of APS: cardiolipin antibody (aCL), lupus anticoagulant (LA), or antibeta 2 glycoprotein I antibodies (ab2GPI).

The neuropsychiatric manifestations of APS are produced by three separate actions of aPLs: disruption of hemostatic reactions at the cell membrane; stimulation of cells leading to an increase in procoagulant activity; and direct neuronal involvement/injury 19. APS may present as psychosis prior to the onset of somatic symptoms 19. Moreover, both APS and SLE can initially present with psychosis, rendering it necessary to include both conditions in the differential diagnosis.

In the present case, APS was characterized by vascular dementia and cognitive impairment, in accordance with previous findings indicating that recurrent strokes can result in multifocal disease and multi‐infarct dementia 19.

Although there are many other causes of psychosis in older adults, no pathognomonic signs that may aid clinicians in distinguishing primary and secondary psychosis have been identified. Primary psychosis remains a diagnosis of exclusion, as approximately 60% of newly diagnosed older adults experience secondary psychosis 24. The risk of psychotic symptoms in older adults may be as high as 23%, especially when dementia is present, as it is a main contributing factor 24. Metabolic, infectious, neurological, and endocrine causes are among the many possible etiologies of late‐life psychosis. However, further studies are required to determining whether further testing in these areas can be used to distinguish primary psychiatric conditions from primary medical/neurological conditions 24. Our findings suggest that specific criteria are required when evaluating older patients and that such criteria should be used in conjunction with clinical, neuroimaging, epidemiological, and neuropsychological information to improve predictive values and narrow the spectrum of possible diagnoses.

Primary psychiatric illnesses that can be considered in the context of a patient with psychotic symptoms include, but are not limited to, schizophrenia, delusional disorder, and major depressive disorder with psychotic features. However, based on the age limits and contradictions between current diagnostic standards and nomenclature used to diagnose psychosis in older patients, the usefulness of the current criteria for this patient population appears controversial.

Corticosteroid use has been reported to induce psychosis in 4.8% of patients with SLE, which can occur at any time during treatment 25, 26. Such adverse effects should be considered when confronting a relapse or poor response to treatment, especially in older adults. Indeed, various complications have been reported in up to 40% of older adults with SLE treated with corticosteroids 27. In a review of patients of all ages receiving treatment with corticosteroids, psychiatric syndromes occurred in a total of 5%, and risk factors for such syndromes included female sex, SLE, and high steroid dose 28.

Our patient's psychotic symptoms resolved after diagnosis and treatment, especially upon administration of rituximab. Although no exacerbations due to corticosteroid treatment were observed, the patient is closely monitored for signs of such adverse effects. Comorbidity of SLE and APS (SAPS) in our patient most likely delayed the initial diagnosis, due to the heterogeneous presentation of both conditions. Moreover, the presence of neurocognitive dysfunction may have obscured the true etiology of her condition, leading clinicians to believe that her psychotic symptoms were indicative of primary disease, rather than a consequence of SAPS.

## Conclusions

This present case underscores the importance of thorough inquiry when psychosis or psychotic features are the main presenting symptoms in older adults, as misinterpretation of various factors may result in delayed diagnosis. Because both SLE and APS may present with neuropsychiatric manifestations, these conditions should be considered when older adults present with first‐time psychotic symptoms. Moreover, the age of the patient, heterogeneous presentation, and mildness of initial symptoms may also contribute to delays in diagnosis. However, it is important to note that primary psychosis should remain a diagnosis of exclusion and that a multidisciplinary approach is key to improve diagnostic success and to reduce morbidity and mortality.

## Authorship

MRG and JRRM: were involved in the patient's care at the Central Military Hospital. PRMO and RBF: wrote the manuscript. LVA: reviewed all MRI images and reports. All authors were involved in the editing and final approval of the manuscript.

## Conflicts of Interest

The authors declare no competing interests.

## Ethical Details

Written consent was obtained from the patient and the family for the publication of this case report and any accompanying images.
